# Desensitization properties of P2X3 receptors shaping pain signaling

**DOI:** 10.3389/fncel.2013.00245

**Published:** 2013-12-06

**Authors:** Rashid Giniatullin, Andrea Nistri

**Affiliations:** ^1^Department of Neurobiology, A. I. Virtanen Institute, University of Eastern FinlandKuopio, Finland; ^2^Department of Neuroscience, International School for Advanced Studies (SISSA), TriesteItaly

**Keywords:** desensitization, extracellular ATP, P2X3 receptor, sensory neuron, pain

## Abstract

ATP-gated P2X3 receptors are mostly expressed by nociceptive sensory neurons and participate in transduction of pain signals. P2X3 receptors show a combination of fast desensitization onset and slow recovery. Moreover, even low nanomolar agonist concentrations unable to evoke a response, can induce desensitization via a phenomenon called “high affinity desensitization.” We have also observed that recovery from desensitization is agonist-specific and can range from seconds to minutes. The recovery process displays unusually high temperature dependence. Likewise, recycling of P2X3 receptors in peri-membrane regions shows unexpectedly large temperature sensitivity. By applying kinetic modeling, we have previously shown that desensitization characteristics of P2X3 receptor are best explained with a cyclic model of receptor operation involving three agonist molecules binding a single receptor and that desensitization is primarily developing from the open receptor state. Mutagenesis experiments suggested that desensitization depends on a certain conformation of the ATP binding pocket and on the structure of the transmembrane domains forming the ion pore. Further molecular determinants of desensitization have been identified by mutating the intracellular N- and C-termini of P2X3 receptor. Unlike other P2X receptors, the P2X3 subtype is facilitated by extracellular calcium that acts via specific sites in the ectodomain neighboring the ATP binding pocket. Thus, substitution of serine275 in this region (called “left flipper”) converts the natural facilitation induced by extracellular calcium to receptor inhibition. Given their strategic location in nociceptive neurons and unique desensitization properties, P2X3 receptors represent an attractive target for development of new analgesic drugs via promotion of desensitization aimed at suppressing chronic pain.

## DESENSITIZATION OF INOTROPIC RECEPTORS

Desensitization is a loss of receptor responsiveness which develops with continuous presence of agonist. Desensitization is a general phenomenon which can be observed in most membrane receptor types, either metabotropic or ionotropic. Unlike metabotropic receptors which desensitize mainly via receptor internalization, thereby temporarily subtracting responsive elements to the extracellular stimulus (Ferguson, 2001), ionotropic receptor desensitization involves structural and functional changes in membrane residing receptors. The fundamental properties of acetylcholine (ACh) ionotropic receptor desensitization were originally described by Katz and Thesleff (1957) working on the frog neuromuscular junction (for review see Giniatullin et al., 2005) and can still be used to supply important principles to understand desensitization of many ionotropic receptors including those activated by extracellular ATP.

Ionotropic ATP receptors comprise P2X1 – P2X7 subtypes widely expressed in various tissues (Burnstock, 2013). The rate of desensitization and recovery processes vary widely within the family of P2X receptors (North, 2002). Thus, desensitization is developing very fast up to complete, yet reversible, loss of response in homotrimeric receptors composed of P2X1 and P2X3 subunits (North, 2002; Coddou et al., 2011). In contrast, the other P2X subtypes are less prone to desensitization. One goal of the present review is to discuss how the strong desensitization process of P2X3 receptors can coexist with the function of P2X3 receptor activation in pain signaling and whether facilitating desensitization might actually be exploited for analgesic purposes. We aim at resolving this conundrum by taking into account the receptor kinetic properties and dynamics.

## P2X3 RECEPTORS UNIQUE PROPERTIES

P2X3 receptors are similar in some desensitization properties (fast onset and slow recovery) to the P2X1 subtype. However, their distinctive characteristics are listed below.

• Fast desensitization onset (ms range; similar time-course for P2X1 subtype)
• Very slow (min range) recovery from desensitization (shared with P2X1 subtype)
• High affinity desensitization (HAD) by very low agonist concentrations without generating a macroscopic current
• Heteromerization of P2X3 with P2X2 subunits to generate sustained non-desensitizing responses
• Remarkable resistance of desensitization onset to changes in temperature and divalent cation concentration
• Strong acceleration of recovery (resensitization) by increases in extracellular Ca^2+^ levels and inhibition by extracellular Mg^2+^
• Speed of recovery highly dependent on temperature (higher temperature, faster recovery)
• Recovery sensitive to signal transduction mechanisms and controlled by the specific modulators

These properties are described in more detail in the following sections of this review.

## DESENSITIZATION ONSET OF P2X3 RECEPTORS

P2X3 receptors can be activated by different agonists such as ATP, α,β-meATP, ATP-γ-S, β,γ-meATP, CTP, 2MeS-ATP and Bz-ATP which can all operate as full agonists (Sokolova et al., 2004, 2006; Pratt et al., 2005; Petrenko et al., 2011, for reviews see North, 2002; Coddou et al., 2011; Fabbretti and Nistri, 2012). Despite their different structures, all these compounds applied in high concentration can generate full size responses that, when recorded as membrane currents in voltage clamp conditions, show very similar fast decay (Sokolova et al., 2006).

**FIGURE 1:**
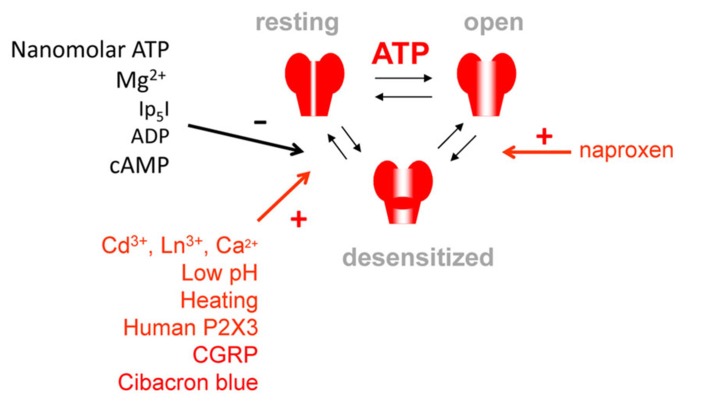
**A simplified kinetic scheme for P2X3 receptor operation indicating resting, open and desensitized receptor states.** Note multiple factors accelerating (+, red arrow) or retarding (-, black arrow) recovery from desensitization, while naproxen promotes desensitization onset. Factors accelerating recovery are expected to facilitate ATP signaling via P2X3 receptor activity, whereas factors retarding recovery (or promoting desensitization onset) could provide the anti-nociceptive effect.

The scheme of **Figure 1** shows that the agonist-bound open receptor can effect a dynamic transition to the closed, desensitized state persisting even when the agonist has come off the receptor. Thus, on P2X3 receptors, as expected from the classical model of desensitization by Katz and Thesleff (1957), the desensitization onset is accelerated by increasing the agonist dose (Sokolova et al., 2006) because the rate of this transition is strongly dependent on the agonist concentration and it, therefore, ensures that a large fraction of the available receptor population dwells in this functionally inactive state. Nevertheless, unlike the phenomenon observed with nicotinic receptors, the onset of P2X3 receptors desensitization is remarkably insensitive to temperature changes (Khmyz et al., 2008). There are only a few experimental manipulations which can specifically interfere with P2X3 receptor desensitization onset. One drug is the anti-inflammatory analgesic naproxen, which is widely used as an anti-migraine agent and which can speed up the desensitization onset of recombinant P2X3 receptors expressed in HEK cells (Hautaniemi et al., 2012, see **Figure 1**). Conversely, acid pH slows down the development of desensitization, although this effect might be indirectly due to reduction of P2X3 receptor potency (Gerevich et al., 2007a).

## SLOW RECOVERY FROM DESENSITIZATION

Restoration of the response after removal of agonist, indicating P2X3 receptor recovery from ATP-induced desensitization, is very slow and can take up to 20 min at room temperature (Cook et al., 1998). This outstanding property makes P2X3 (and P2X1) receptors very different from other ligand-gated receptors (Cook et al., 1998). Even though the recovery occurs very slowly, it proceeds to full return of P2X3 receptor response: this reversibility is a useful criterion to distinguish desensitization from the irreversible rundown which is a common feature of many ionotropic receptors including the P2X1 subtype (Lewis and Evans, 2000).

We have previously shown that recovery of P2X3 receptors from desensitization is an agonist-specific process (Sokolova et al., 2004). It can be very fast for agonists like β,γ-meATP, but unusually slow for 2MeS-ATP (Sokolova et al., 2006). Furthermore, recovery displays distinctive properties from desensitization onset because, in addition to its time-course, it is modulated by factors such as temperature (Khmyz et al., 2008), cibacron blue (Alexander et al., 1999), extracellular Ca^2+^ (Cook et al., 1998) and others listed in **Figure 1**. The independent modulation of desensitization onset and recovery suggests that they are likely to have different determinants (see details in the “Structural Determinants of Desensitization”).

## HIGH AFFINITY DESENSITIZATION

Apart from the “classical” desensitization arising from the open receptor state, there is an additional process of slow onset desensitization called “HAD” – that is, a low nanomolar concentration of the agonist inhibits P2X3 receptor responses without evoking a macroscopic current (McDonald et al., 2002; Sokolova et al., 2004, 2006; Pratt et al., 2005). We have shown that this phenomenon is also agonist-specific, being particularly strong for the natural agonist ATP (Sokolova et al., 2004, 2006). Thus, ATP is about a 10-fold more potent inducer of HAD than α,β-meATP (IC_50_ 2 and 20 nM, respectively), even though both agonists have almost the same EC_50_ values (Sokolova et al., 2004).

Given that the ambient level of extracellular ATP in various tissues is in the nanomolar range (Kuzmin et al., 1998; Lazarowski et al., 2003), one might suggest that a fraction of P2X3 receptors is continuously inactivated *in vivo*. Such inactivation could largely reduce the ability of P2X3 receptor expressing neurons to detect and generate nociceptive signal in response to acute ATP release. Furthermore, diadenosine polyphosphates, that are endogenously produced by bridging two adenosine moieties with a chain of two or more phosphates, can act as powerful HAD inducers (McDonald et al., 2002). This observation widens the range of substances that can modulate desensitization of P2X3 receptors and emphasizes the functional role of HAD as discussed in detail in the present review.

### P2X3 ACTIVITY IS FACILITATED BY EXTRACELLULAR CALCIUM AND INHIBITED BY MAGNESIUM

One outstanding feature of the P2X3 receptor is its unusual sensitivity to extracellular Ca^2+^ which is specifically targeting receptor desensitization. The exciting finding of the facilitatory action of extracellular Ca^2+^ on P2X3 receptor was first described by McClesky’s group (Cook et al., 1998). Interestingly, Ca^2+^ selectively and positively controls the recovery stage of receptor desensitization without changes in desensitization onset (Giniatullin et al., 2003). The most intriguing finding is that there is a sort of “memory” of the receptor system which remains in the facilitated state for several mins after the initial *transient* contact with the divalent cation (Cook et al., 1998). Such resensitization by Ca^2+^ on P2X3 receptor is functionally opposite to the well documented inhibitory action of Ca^2+^ on other P2X receptor subtypes (Virginio et al., 1998; Ding and Sachs, 2000). In fact, in the case of P2X2 receptors, extracellular Ca^2+^ rather promotes desensitization (Ding and Sachs, 2000).

We have previously observed that, on native P2X3 receptors, extracellular Mg^2+^ can produce an opposite effect to Ca^2+^ (Giniatullin et al., 2003). Thus, Mg^2+^ delays receptor recovery from desensitization, while the onset of desensitization remains unchanged. Ca^2+^-free solution has the similar inhibitory action on receptor resensitization. As the modulatory effects were observed with physiological concentrations of Ca^2+^, these finding outline a potential role of this mechanism even *in vivo*.

Our recent study has demonstrated that a single aminoacid (S275) within the left flipper region of the P2X3 receptor ectodomain is a likely determinant for the facilitatory action of extracellular Ca^2+^ (Petrenko et al., 2011). Thus, in the A275 mutant, extracellular Ca^2+^ induces an inhibitory effect on P2X3 receptor mediated responses instead of the facilitation normally seen with wildtype (WT) receptors.

## UNUSUALLY HIGH TEMPERATURE SENSITIVITY

Another unexpected desensitization property of P2X3 receptors was described in detail by Krishtal’s group. They showed that, while the desensitization onset is almost temperature insensitive, the recovery process is very temperature sensitive with a Q_10_ coefficient of about 10 (Khmyz et al., 2008). The temperature sensitivity of HAD is also high but clearly smaller than the one of the recovery process (Khmyz et al., 2008). These findings suggest that, unlike most *in vitro* experiments performed at room temperature, the recovery process at normal body temperature is much faster and that, in physiological conditions, the probability of repeated receptor activation is strongly enhanced.

Interestingly, the recycling of P2X3 receptors in the peri-membrane region measured by the TIRF/FRAP technique also shows very high sensitivity to temperature (Pryazhnikov et al., 2011).

## KINETIC MODELING OF P2X3 RECEPTOR DESENSITIZATION

A formal description of P2X3 receptor behavior using kinetic modeling allows the exploration of silent receptor conformations, prediction of new receptor desensitization properties, and provides a mechanistic explanation for experimentally observed phenomena. Modeling of P2X3 receptor kinetics clearly indicates that a cyclic scheme of receptor reversible transitions modified from the original proposal by Katz and Thesleff (1957) is the most suitable to account for the experimental data which cannot be adequately explained with linear or bifurcation models (Sokolova et al., 2006). This model integrates all the main steps of P2X3 function such as binding, gating and desensitization, which are presented in an over-simplified form in **Figure 1**.

In the complete receptor model with full activation caused by three agonist molecules bound to it, the receptor transition into the desensitized state occurs mainly from the open state. Furthermore, this model can simulate HAD, implying the existence of a single molecule bound desensitized state with high agonist affinity (Sokolova et al., 2006). As a result, the model fully reproduces all the main properties of the P2X3 receptor, including fast desensitization, slow recovery and HAD.

The rate limiting role of agonist dissociation from the desensitized state for resensitization obtained with this theoretical approach accords with agonist unbinding from P2X3 receptors measured using radiolabeled ATP (Pratt et al., 2005). Most importantly, the kinetic model predicts that desensitization, being the next step after binding of ATP and channel opening, should depend not only on “intrinsic” desensitizing properties of the P2X3 receptor, but also on agonist binding and channel gating. This view is essential when trying to find out and analyze the numerous data on structural determinants of desensitization.

To explain P2X3 receptor operation and in particular HAD, Karoly et al. (2008) have subsequently proposed an allosteric model that retains the cyclic scheme of Sokolova et al. (2006), but it adds to it an additional transition, namely a receptor open state occurring when two rather than three ATP molecules are bound. Thus, Karoly et al. (2008) argue that their revised model is the simplest way to explain increased affinity of non-occupied binding sites when receptors are partially occupied. Future biophysical studies using microscopic recording of single channel currents may be necessary to clarify this proposal.

## STRUCTURAL DETERMINANTS OF DESENSITIZATION

At membrane level functional homomeric P2X3 receptors are assembled as trimers, whereby each subunit is composed of a large extracellular loop containing the ATP binding domain, two transmembrane domains and intracellular N- and C- termini (for reviews see Khakh, 2001; Khakh and North, 2006; Kawate et al., 2009, Fabbretti and Nistri, 2012). This complex structure has been extensively probed to find out the molecular determinants of desensitization.

One pioneer study showed that, using recombinant receptors expressed in Xenopus oocytes, chimeric P2X2 receptors containing P2X1 or P2X3 domains acquire strong desensitization, while chimeric P2X1 or P2X3 receptors with P2X2 domains show very weak desensitization (Werner et al., 1996). The domains necessary to alter the desensitization phenotype include the most hydrophobic segments of the molecules, which are thought to be membrane-spanning segments (Werner et al., 1996). These findings led Werner et al. (1996) to propose that desensitization requires the interaction of these receptor long segments (comprising 34 aminoacids).

Many subsequent studies were focused on finding key residues determining the desensitization process of P2X receptors. Thus, it has been shown that certain C-terminus residues are important to express the slow desensitization of P2X2 receptors (Koshimizu et al., 1998; Smith et al., 1999). Likewise, the N-terminus half of the P2X3 receptor ectodomain plays a role in the slow recovery from desensitization (Zemkova et al., 2004).

Our previous study has identified several residues in the ectodomain which determine the desensitization properties including Glu111, Asp220, and Asp266 (Fabbretti et al., 2004). By site-directed mutagenesis to alanine, it was possible to observe a pleiotropy of receptor responses like fast onset combined with either faster or slower recovery (Glu111Ala and Asp220Ala, respectively), or very slow onset (resembling the non-desensitizing P2X2 receptor) combined with fast recovery (Asp266Ala). These various combinations of kinetic properties provide additional evidence for the existence of independent determinants of desensitization onset and recovery.

Our *in silico*exploration of the P2X3 receptor molecular model predicted that S275 within the ectodomain region termed *left flipper* as a contributor to ATP binding and suggested that its manipulation would result in changes in the desensitization properties (Petrenko et al., 2011). Indeed, substituting S275 with alanine produces a mutant with slow desensitization onset, fast resensitization and lack of HAD (Petrenko et al., 2011). In this experiment, HAD remained minimal even with higher agonist concentrations (to compensate for reduced potency), suggesting that this residue is indeed important to express the inhibitory action of low nanomolar concentrations of ATP. Substituting S275 with more hydrophobic aminoacids further slows down desensitization onset and accelerates recovery, indicating these two properties to be reciprocally interrelated (Petrenko et al., 2011).

**FIGURE 2:**
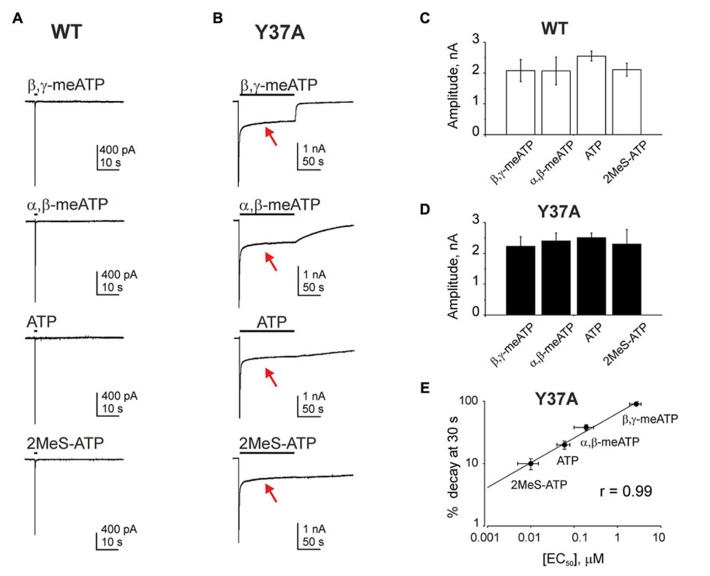
**The action of different agonists on the WT and Y37A receptor.** **(A)** Currents activated by 2 s application of β,γ-meATP, α,β-meATP, ATP, or 2MeS-ATP (all 100 μM) on WT receptors. **(B)** Currents activated by 2 min application of β,γ-meATP, α,β-meATP, ATP, or 2MeS-ATP (in keeping with higher potency, all at 10 μM) on Y37A mutant receptors. Note similar peak amplitudes and plateau-like components (red arrows) but different current decays during the washout phase. **(C,D)** Histograms showing the mean amplitudes of peak currents induced by different agonists on WT **(C)** or Y37A receptor **(D)**. **(E)** Graph showing a correlation between differential agonist-specific current decays (measured after 30 s of washout) and corresponding EC_50_ values for each agonist. Datapoints from 3 to 12 cells. Reproduced with modifications from Jindrichova et al. (2011) with permissions from John Wiley and Sons.

Manipulations of the transmembrane P2X3 domains have indicated additional contributors to shape desensitization. Thus, substitution with alanine (or phenylalanine) of the highly conserved Y37 yields initial fast desensitization with a large residual current, remarkably resistant to further desensitization (Jindrichova et al., 2011; see **Figure 2A**). Unlike the WT, this long lasting persistent current (highlighted by red arrow in **Figure 2B**) is systematically observed in the Y37A mutant in the presence of four different agonists (**Figure 2B**) applied at saturating concentrations that induce analogous amplitude of membrane current (**Figures 2C,D**). The latter indicated that this unusual plateau-like current is independent from the nature of the agonist. In sharp contrast, at the end of the agonist application, a highly variable (agonist-specific) rate of deactivation is observed (**Figure 2B**). This variable deactivation is related to the EC_50_ values for each agonist (**Figure 2E**). Furthermore, the rate of deactivation correlates also with the rate of agonist dissociation from the desensitized receptor state (fast deactivation, fast dissociation, see Sokolova et al., 2006). Kinetic modeling may help to explain these unusual phenomena by assuming facilitated re-opening of the P2X3 channels from the desensitized to the conducting states (Jindrichova et al., 2011).

In conclusion, current evidence shows multiple determinants of desensitization throughout the whole receptor structure (ectodomain, transmembrane and intracellular termini).

Useful clues to the biophysical nature of desensitization may also come by comparing P2X3 receptors with sister receptors containing other P2X subunits. Thus, using voltage clamp fluorometry applied to the P2X1 subtype, which is also prone to fast desensitization, Lörinczi et al. (2012) have proposed that the cys-rich *head* domain of this receptor is involved in both channel activation and desensitization. In the P2X1 subtype, high sensitivity to low doses of ATP is also reported, although desensitization in this case develops after detectable membrane currents (Rettinger and Schmalzing, 2003). Despite the fact that P2X7 receptors have low affinity for their natural ligand ATP (active at mM concentrations), ongoing desensitization has also been proposed to exist in this subtype, yet masked by the overlapping process of ion pore widening (Khadra et al., 2013).

## FUNCTIONAL ROLE OF DESENSITIZATION OF P2X3 RECEPTORS

Nowadays there is strong evidence that P2X3 receptors participate in chronic pain, a most distressing clinical state often resistant to treatment (Burnstock, 2001; North, 2004). In fact, P2X3 channels are almost exclusively expressed by nociceptive neurons (Chen et al., 1995; Lewis et al., 1995), and impaired pain-evoked responses (especially of inflammatory type) are observed in P2X3 knockout mice (Cockayne et al., 2000; Souslova et al., 2000). These data accord with earlier reports that injection of α,β-meATP into the rat paw evokes strong nociceptive behavior which is paradoxically prevented by a previous application of the same agonist (Bland-Ward and Humphrey, 1997), pointing to a functional role of P2X3 receptor desensitization in shaping pain responses *in vivo*. However, in view of the fast desensitization of P2X3 receptors and the high likelihood of their HAD because of ambient ATP (and its metabolites), one may wonder how to reconcile such characteristics with a functional role of these receptors in sustained pain signaling (North, 2004).

One possible explanation is that, in healthy subjects, the main role of HAD in ATP-gated P2X3 receptors is to prevent inappropriate excitation of nociceptive pathways (and associated pain sensitivity) when there is a high probability of ATP release from surrounding tissues. In other words, HAD might be viewed as an intrinsic mechanism of “anti-nociception” in normal states, working in cooperation with fast desensitization to restrict ATP-mediated signaling. This protective process might be important in conditions like physical exercise when there is a continuous release of ATP in muscles to the level comparable with EC_50_ of P2X3 receptors (Hellsten et al., 1998; Mortensen et al., 2011).

Assuming a constitutive inhibition of P2X3 receptor activity by ambient purines, only strong, burst-like ATP release can perhaps represent a stimulus large enough to overcome any intrinsic anti-nociceptive effect (Giniatullin et al., 2008). In contrast, in chronic pain in man, particularly of inflammatory origin, P2X3 receptors might play a direct pro-nociceptive role (Burnstock, 2001) because the desensitization properties of P2X3 receptors could be modified by several cellular mechanisms that collectively or individually promote nociceptive sensitization (**Figure 1**):

(i) removal of ambient ATP via extracellular NTDases;
(ii) decreased HAD;
(iii) accelerated recovery from desensitization;
(iv) heteromerization with non-desensitizing P2X2 receptors.

Although the *first mechanism* of sensitization has not been studied in relation to pain, its feasibility is indirectly supported by experiments on taste buds (Vandenbeuch et al., 2013). In fact, recent studies have shown expression of P2X3 receptors (along with P2X2 ones) in nerves supplying the taste buds (Finger et al., 2005). Using the NTDase-2 knockout mouse, it has been observed that excessive levels of extracellular ATP generated in the taste bud could inactivate nerve terminal P2X receptors and block the taste response by promoting desensitization via a phenomenon resembling HAD (Vandenbeuch et al., 2013). Thus, the extent of breakdown and clearance of extracellular ATP may be important to regulate nociceptive purinergic signaling.

As for the *second mechanism*of sensitization, evidence clearly shows that resensitization of P2X3 receptors *in vivo*is much faster than *in vitro* (Khmyz et al., 2008), and may be expected to occur even faster if local inflammatory reaction raises tissue temperature. Furthermore, inflammatory cells like macrophages releasing TNFα can further upregulate P2X3 receptor activity (Franceschini et al., 2012, 2013). It is also noteworthy that human P2X3 receptors recover about twice faster than analogous rodent receptors (Pratt et al., 2005). Finally, the P2X3 receptor recovery may be accelerated, via release of neuropeptides and neurotrophins (Fabbretti et al., 2006; D’Arco et al., 2007) and by extracellular acidification (Gerevich et al., 2007a) which occurs in inflammation and cancer (Tannock and Rotin, 1989). Thus, the migraine mediator CGRP not only largely increases the amplitude of P2X3 currents generated in nociceptive trigeminal neurons, but it also significantly speeds up recovery from desensitization via an intracellular kinase-dependent process of P2X3 receptor phosphorylation (Fabbretti et al., 2006; Giniatullin et al., 2008).

The possibility of the *third mechanism* of sensitization via reduced HAD is supported by decreased inhibitory action of nanomolar ATP at higher temperature (Khmyz et al., 2008).

Finally, enhanced expression of heteromeric P2X2/3 receptors producing typically non-desensitizing plateau currents might be the *fourth mechanism* of sensitization contributing to nociceptive signaling (North, 2004). However, recent study revealed the prevailing expression of P2X3 subunits in dorsal root ganglion neurons in primates, including human sensory neurons (Serrano et al., 2012). Thus, the contribution of P2X2/3 receptors to chronic pain is probably more important at the level of nociceptive pathways within the spinal dorsal horn as indicated by the inhibition of nociceptive neuron firing by A-317491, a potent P2X2/3 antagonist (Xu et al., 2012).

A proposal for the physiological role of desensitization should also include mechanisms which augment this inhibitory process. Indeed, recovery from desensitization can be delayed via GPCR signaling (**Figure 1**) involving P2Y receptors (Gerevich et al., 2007b), GABA_B_ receptors (Sokolova et al., 2003) and unidentified membrane receptors activated by extracellular cAMP (Mamenko et al., 2010). These mechanisms may be additional to the intrinsic inhibition of P2X3 receptor activity via C-terminal Src inhibitory kinase (Csk)-mediated tyrosine phosphorylation of the receptor (D’Arco et al., 2009). Future studies should address whether in chronic pain models there is a disruption in the intricate molecular mechanisms regulating P2X3 expression and function.

## ANALGESIC DRUGS PROMOTING DESENSITIZATION OF P2X3 RECEPTORS

### INFLAMMATORY AND NEUROPATHIC PAIN

The anti-nociceptive action of the selective P2X3 antagonist A-317491 in inflammatory and neuropathic pain was first reported in a study aimed at counteracting chronic pain in animal models (Jarvis et al., 2002). The traditional pharmacological approach to anti-nociception is usually based on the development of receptor antagonists with good bioavailability, high specificity, and minimal side-effects. One such example is a novel selective P2X3/P2X2/3 receptor antagonist AF-353 which is orally bioavailable (Gever et al., 2010). Other potential analgesic agents with different structure based on P2X3 receptor antagonism are reviewed by Gum et al. (2012) and by A. P. Ford in this series. Some of these compounds are already investigated in clinical trials, although none of them has yet received statutory approval by regulatory agencies for human use (Ford, 2012). A new line in drug development for the prevention of P2X3 mediated pain should target facilitation of desensitization. In fact, Bland-Ward and Humphrey (1997) have previously shown that an analgesic effect could be induced by injection of the full P2X3 agonist, α,β-meATP after an initial pro-nociceptive effect. More promising clues for pain suppression treatment may, however, come from the use of promoters of desensitization which show little or no agonist activity. Thus, purotoxin-1, a peptide recently isolated from the Asian wolf spider Geolycosa, powerfully inhibits P2X3 receptors by strongly promoting their desensitized state and provides effective anti-nociception in various pain models *in vivo* (Grishin et al., 2010) while the antagonist P1, P5-di[inosine-5^′^] pentaphosphate binds to the desensitized state of the P2X3 receptor in DRG neurons (Ford et al., 2005).

### MIGRAINE AND TRIGEMINAL PAIN

Mg^2+^ can hardly be considered as a selective anti-migraine agent, and is typically known to be the endogenous blocker of NMDA channels (Nowak et al., 1984). There are, however, repeated suggestions to include this agent in the complex therapy of trigeminal migraine pain (this subject has been extensively discussed by Pardutz and Vecsei, 2012; and Mauskop and Varughese, 2012). As Mg^2+^ inhibits desensitizing ATP-evoked currents in rat cultured sensory neurons (Giniatullin et al., 2003), this effect might be considered to contribute to its analgesic action *in vivo* (Crosby et al., 2000). Given that Mg^2+^ specifically interferes with desensitized receptor states, one could expect the use-dependent anti-nociceptive action of this divalent cation in pain mediated by high concentrations of ATP acting on the P2X3 receptor. Of course, modulation by Mg^2+^ of P2X3 receptor activity may be complementary to the divalent cation role in NMDA receptor function that is thought to be dysregulated in trigeminal ganglia and underlying pain sensitization (Laursen et al., 2013).

The high expression of P2X3 receptors by the vast majority of trigeminal sensory neurons (Simonetti et al., 2006) provides the molecular substrate for P2X3 mediated trigeminal pain including migraine (Giniatullin et al., 2008). In experimental model of trigeminal pain *in vitro*, expression of P2X3 membrane receptors by trigeminal neurons is amplified by the migraine mediator CGRP that operates via intracellular kinases and enhanced P2X3 receptor trafficking from intracellular compartments (Fabbretti et al., 2006). Likewise, in transgenic mice expressing human mutated P/Q calcium channels as observed in Familial Hemiplegic Migraine type 1, the activity of P2X3 channels is constitutively enhanced (Nair et al., 2010) probably because of the higher background release of CGRP as shown by the phenotype reversal evoked by a CGRP receptor antagonist (Hullugundi et al., 2013). To further support the role of PX3 receptors in migraine pain, there are data obtained with well-established anti-migraine drugs. Thus, naproxen, a popular anti-headache analgesic, directly inhibits P2X3 receptors by facilitating receptor desensitization (**Figure 1**), an inhibitory effect that is potentiated in the presence of the algogen nerve growth factor (Hautaniemi et al., 2012), the level of which is elevated in patients with chronic migraine (Jang et al., 2011).

### CANCER PAIN

Accumulating evidence suggests the involvement of P2X3 receptors in bone tissue cancer pain. The bone has a very specific environment including diverse cell populations and a specialized hormonal control by parathyroid hormone and parathyroid hormone-related protein which regulate Ca^2+^ release from osteoclasts (Soki et al., 2012). Thus, in bone cancer there is a massive release of Ca^2+^ leading eventually to hypercalcaemia which as mentioned above is a potent and unique mechanism to facilitate, via resensitization, the function of P2X3 receptors. In some pathological conditions, for instance, during parathyroid hormone-related protein-mediated hypercalcemic crisis, the serum Ca^2+^ can raise from normal level of ~1.5 mM up to 4.8 mM (Rahil and Khan, 2012). There can be also malignancy-associated hypercalcemia, often associated with headache when the level of Ca^2+^ released from osteoclasts is rising locally within the bone (Basso et al., 2011). When the intense ATP release coincides with local enhancement of extracellular Ca^2+^ this could result in a strong nociceptive firing through increased activity of P2X3 receptors. The antagonism of Ca^2+^ effect on P2X3 receptor by Mg^2+^ (Giniatullin et al., 2003) can provide a rationale for the high analgesic efficiency of the latter in cancer pain (Crosby et al., 2000). Thus, the selective P2X3, P2X2/3 receptor antagonist A-317491 transiently attenuates cancer-induced bone pain in mice, but has no effect at the late stage of bone cancer (Hansen et al., 2012). Bone cancer pain in rats is reduced by the blockade of P2X3 and P2X2/3 receptors with AF-353 (Kaan et al., 2010). Furthermore, there is an increased expression of P2X3 receptors in CGRP immunoreactive nerves during tumor growth suggesting their role in cancer-related pain (Gilchrist et al., 2005). Likewise, P2X3 receptors are functionally up-regulated in dorsal root ganglion neurons of a rat model of bone cancer (Wu et al., 2012).

## CONCLUSION

Desensitization of nicotinic ACh receptors is now considered an important process for neuronal signaling in health and disease (Giniatullin et al., 2005). In fact, there are ongoing efforts to develop drugs (“silent desensitizers”; Buccafusco et al., 2009) to modulate cholinergic function via receptor desensitization. The path is, therefore, open to look for chemical agents to desensitize P2X3 receptors selectively and to discover their impact on physiological or pathological conditions.

## Conflict of Interest Statement

The authors declare that the research was conducted in the absence of any commercial or financial relationships that could be construed as a potential conflict of interest.

## AUTHOR CONTRIBUTIONS

Rashid Giniatullin and Andrea Nistri contributed to the writing of this review.
